# Enhanced Growth and Activities of the Dominant Functional Microbiota of Chicken Manure Composts in the Presence of Maize Straw

**DOI:** 10.3389/fmicb.2018.01131

**Published:** 2018-05-29

**Authors:** Lili Zhang, Lijuan Li, Xiaoguang Pan, Zelu Shi, Xihong Feng, Bin Gong, Jian Li, Lushan Wang

**Affiliations:** ^1^State Key Laboratory of Microbial Technology, Shandong University, Jinan, China; ^2^School of Computer Science and Technology, Shandong University, Jinan, China; ^3^Lu Qing Seed Co., Ltd., Jinan, China; ^4^Laboratory of Antimicrobial Systems Pharmacology, Monash Biomedicine Discovery Institute, Department of Microbiology, Monash University, Melbourne, VIC, Australia

**Keywords:** chicken manure compost, metaproteomics, functional microbiota, microbial diversity, *Bacillus*

## Abstract

As a consequence of intensive feeding, the bulk deposition of livestock manure causes severe environmental problems. Composting is a promising method for waste disposal, and the fermentation process is driven by microbial communities. However, chicken manure contains diverse gut microbes, mainly species derived from *Proteobacteria*, which may include pathogens that threaten human health. To evaluate composting as a harmless treatment of livestock manure, the dynamics of the microbiota in two chicken manure composts were studied, and the influences of adding maize straw on the compost microbiota were compared. The results revealed that microbes from *Firmicutes* including *Bacillus* and *Lentibacillus* are the most dominant degraders with a strong amino acid metabolism, and they secrete a diverse array of proteases as revealed in metaproteomics data. The addition of maize straw to the chicken manure compost accelerated species succession at the initial stage, and stimulated carbohydrate metabolism in the dominant microbiota. Besides, under the resulting high temperature (>70°C) conditions, the relative abundance of *Proteobacteria* was reduced by 78% in composts containing maize straw by day 4, which was faster than in compost without added maize straw, in which the abundance was reduced by 66%. Adding maize straw to chicken manure composts can therefore increase the fermentation temperature and inhibit the growth of *Proteobacteria*. In general, these findings provide increased insight into the dynamic changes among the dominant functional microbiota in chicken manure composts, and may contribute to the optimization of livestock manure composting on an industrial scale.

## Introduction

The animal breeding industry has expanded rapidly in recent years across the globe due to the increasing demand for animal products, and this has resulted in an increase in livestock-derived manure. The bulk deposition of livestock manure causes severe environmental problems including offensive odors, soil, and water pollution, and the spread of antibiotic resistance, which is a great threat to human health (Nicholson et al., 2005; Burton, 2007). It is therefore crucial to find an effective biosafety method for processing livestock manure.

Composting can transform organic agricultural and industrial wastes into bio-fertilizers in an environmentally friendly way, and is a promising method for waste disposal (Bertoldi et al., 2014). During the composting process, microbial communities play an important role in the degradation of organic materials. To better understand the composting process and increase the efficiency of composting, the dominant functional microbial communities and their dynamics need to be investigated. However, to date, most studies have focused on the composition of the microbial communities in composts, while their functions and dynamics have remained largely unexplored (Bernal et al., 2009; Hui et al., 2012; Gannes et al., 2013; Wei et al., 2014). Fortunately, the emerging discipline of metaproteomics can address this issue, since it probes the functions of proteins *in situ* (Johnson-Rollings et al., 2014). Furthermore, due to the relatively longer half-life of proteins compared with RNA, and the lack of requirement for amplification by PCR-based methods, metaproteomics data may be more readily obtainable and reliable than metatranscriptomics and metagenomics data (Urich et al., 2014; Vanwonterghem et al., 2014). In addition, by combining high-throughput sequencing and metaproteomics approaches, the composition and functions of microbial communities can be linked, and dominant functional members that make the most important contributions to the composting process can be identified.

In our previous studies, we employed integrated meta-omics experiments comprising high-throughput sequencing technology and metaproteomics based on native polyacrylamide gel electrophoresis (PAGE), gelatin zymography, and liquid chromatography-tandem mass spectrometry (LC-MS/MS) to successfully characterize the functional microbial communities in lignocellulosic composts (Zhang et al., 2015a,b, 2016). However, compared with plant biomass, the main component of which is lignocellulose, livestock manure has a much more distinct composition (Petersen et al., 2007). For example, chicken manure is produced by chickens that mainly feed on cereals and beans, and hence it has a high protein content. The functions of the microbiota in chicken manure composts are therefore likely to be different from those of the microbiota in lignocellulose-based composts. The low carbon-to-nitrogen ratio (C/N) of chicken manure can also support the growth of microbial pathogens belonging to the bacterial phylum *Proteobacteria* (Hu et al., 2016). Furthermore, large amounts of antibiotics are often added to animal feed in an attempt to control disease in intensive industrial systems (Yang et al., 2013). Thus, a comprehensive understanding of the dominant functional microbial communities and their succession in chicken manure composts is highly desirable.

The goal of the present study was to determine the dominant functional microbial communities in livestock manure composts, and probe their dynamics. To achieve this goal, chicken manure was used as a compost feedstock, and the composition and functional dynamics of the dominant microbial communities were investigated. Besides, adding crop straw like maize straw as bulking agent into livestock manure composts could increase air permeability of the compost, adjusting C/N, therefore promotes the growth of microbial communities. Hence, to examine the influence of the C/N ratio on the structure of the compost communities, microbial population dynamics were investigated following the addition of maize straw.

## Materials and Methods

### Compost Performance and Sampling

Chicken manure applied in this study was broiler manure, and was collected from chicken farms surrounding Jinan in China and used for composting in July, 2017. Two chicken manure composts were constructed. One pile of chicken manure compost was supplemented with fresh maize straw shredded into ∼5-cm pieces to achieve a C/N of 25-35, and the other pile with no addition was set as the control. Composting was performed as described previously (Zhang et al., 2015a). Briefly, both compost piles were 10 m long, 3 m wide, and 1.5 m high, and samples were removed at days 0, 4, 8, 16, 24, 32, and 40. For each pile, triplicate samples containing approximately 500 g of material were collected from three different places in the middle layer (20-50 cm from the surface of the compost), and were stored at -20°C until needed. A total of 42 samples were taken at different composting times (Supplementary Table S1). Samples taken from chicken manure compost are referred to as CM0–40, and samples collected from chicken manure compost with maize straw added are referred to as CS0–40 (where the number indicates the sampling day). Three triplicate samples from the same pile are referred to as “-1”, “-2”, “-3.”

### Physicochemical Properties and Zymography Analysis

The temperature, pH, and electrical conductivity (EC) were determined essentially as described previously (Zhang et al., 2015a). Briefly, during each sampling time, the temperatures of the sampling sites and the environment were measured with a thermometer (TES1310, TES, Taiwan, China). For all 42 samples, a water extract with a compost-to-distilled water ratio of 1:9 (w/v) was measured using a pH meter (Sartorius, Göttingen, Germany) and a conductivity meter (DDSJ-308A, INESA, Jiangxi, China) for pH and EC determination, respectively. The pH and EC determination experiments were conducted in triplicate and averaged to obtain the final results.

Crude enzymes were extracted as described by Yu et al. (2007). Each treatment was replicated three times. Gelatin zymography was performed in sodium dodecyl sulfate (SDS)-containing polyacrylamide gels using a modification of the method described by Tsujii et al. (1997). Briefly, crude enzymes were separated on 13.5% SDS-PAGE with 0.1% gelatin incorporated at 4°C. After electrophoresis each gel was washed two times with 2.5% Triton X-100 for 20 min, and then the gel was washed two times with 2.5% Triton X-100/0.6% Tris to remove the SDS, transferred to a bath containing 0.6% Tris (pH 8.0), and incubated at 70°C for 2 h. Subsequently, 0.5% Coomassie blue was used to fix and stain the gel for 1 h, and the gel was destained in 10% acetic acid/10% ethanol and visualized using a BenQ scanner 7550R (BenQ Corporation, Taipei, China).

### High-Throughput Pyrosequencing

A PowerSoil DNA Isolation Kit (MO BIO Laboratories, Carlsbad, CA, United States) was used for the extraction of DNA from compost samples according to the manufacturer’s instructions. For each sample, triplicate subsamples from three extractions were mixed to obtain a representative DNA sample. The concentration of the obtained DNA was measured at 260 nm using a NanoDrop ND-1000 spectrophotometer (Thermo Fisher Scientific Inc., Waltham, MA, United States), and samples were stored at -80°C until needed. Pyrosequencing was performed as previously described (Zhang et al., 2015a) except that the barcoded fusion primers were replaced with 338F (5′-ACTCCTACGGGAGGCAGCAG-3′) and 806R (5′-GGACTACHVGGGTWTCTAAT-3′) for the amplification of 16S rRNA genes. The PCR conditions for amplification were 95°C for 3 min, followed by 27 cycles of 95°C for 30 s, 55°C for 30 s, and 72°C for 45 s, followed by a final elongation step at 72°C for 10 min. The amplicons were pooled in equimolar concentrations and sequenced using the Roche GS FLX+ system. A Quantitative Insights into Microbial Ecology (QIIME) pipeline was applied to control the data quality, including length-based filtering and read-quality filtering. Normalization of sample reads was performed by random selection closed to the lowest data sets (19,381 reads for each compost sample) to compare data sets and eliminate biases.

### Taxonomic Analysis

USEARCH version 7.1 software^1^ was used for read clustering, and the identity cutoff for operational taxonomic unit (OTU) clustering was set at 0.03. The UCHIME method was used to remove chimeric OTUs from further analysis. Taxonomy assignment was performed with the Ribosomal Database Project (RDP) classifier Bayesian algorithm to analyze clustered OTUs against the 16S reference database Silva^2^. QIIME pipeline was used to perform the computation of the Chao1 metric and other indices. PICRUSt was applied to predict the functions of compost communities based on the 16S rRNA gene library composition (Langille et al., 2013).

### Metaproteomics and Bioinformatics

The metaproteome of the samples was extracted as described by Zhang et al. (2015a). Briefly, to extract the proteins, a mixture of a 100-g sample with 400 mL sterile water was stirred overnight at 4°C. Following centrifugation at 10,000 ×*g* at 4°C for 10 min, the collected supernatant was filtered through a 0.22-μm membrane and further ultrafiltered with a 3-kDa cut-off membrane (Sigma-Aldrich, NJ, United States). Trichloroacetic acid was used to precipitate the concentrated proteins, and the precipitate was resuspended in double-distilled water. Then 50 μg of protein solution was washed with 50 μL of degeneration buffer [0.5 M Tris–HCl, 2.75 mM ethylenediaminetetraacetic acid (EDTA), and 6 M guanidine-HCl, Sigma-Aldrich] and incubated at 37°C for 2 h after the addition of 30 μL of 1 M dithiothreitol (Sigma-Aldrich). After the addition of 50 μL of 1 M iodoacetamide (Sigma-Aldrich), alkylation of the mixture was performed by keeping it in the dark for 1 h. After centrifugation, the pellet was digested with trypsin at a ratio of 1: 50 (w/w, trypsin:centrifuged proteins) and stirred overnight at 37°C. A Millipore ZipTip C18 column (Sigma-Aldrich) was applied for desalination, and the peptides were dissolved in 10 μL of elution buffer containing 50% (v/v) acetonitrile (ACN) and 0.1% (v/v) trifluoroacetic acid. The eluted peptides were subjected to LTQ-Orbitrap-MS/MS at Shandong University of Shandong Province, China, with a Prominence nano LC system (Shimadzu) coupled to an LTQ-Orbitrap Velos Pro ETD mass spectrometer (Thermo Fisher Scientific). In the linear ion trap, the 10 most intense precursor ions over the threshold of 5,000 counts were chosen for MS/MS analysis. To prevent repetitive selections of peptides, dynamic exclusion within 60 s was set.

Proteome Discovered software 1.4 (Thermo Fisher Scientific) with the SEQUEST search engine was used for the alignment of the MS/MS spectra. The genera with relative abundances in the top 20 at each sampling time were used as reference databases. Oxidation and carbamidomethylation were set as the dynamic modification and static modification, respectively. Search results were filtered by automatic decoy database searches with a false discovery rate set as 0.05. If a probability <0.05 was achieved and at least two peptides were identified (*q*-value < 0.05), the protein identifications were accepted. For a protein to be considered valid, the protein was required to be identified in at least two replicates. SignalP 4.1 Server was applied to classify signal peptide sequences.

The software Origin5.0 was used to generate the figure of physicochemical properties. Figures of community structure were conducted by the software R. The software Matlab (version R2014a) was used to conduct the statistical analysis and generate the Heatmap figure of metaproteomics.

### Accession Numbers

Sequencing data generated in this study have been deposited at the NCBI Sequence Read Archive under accession codes SRP117004 and SRP117012.

## Results

### Physicochemical Analysis of Chicken Manure Compost Samples

**FIGURE 1:**
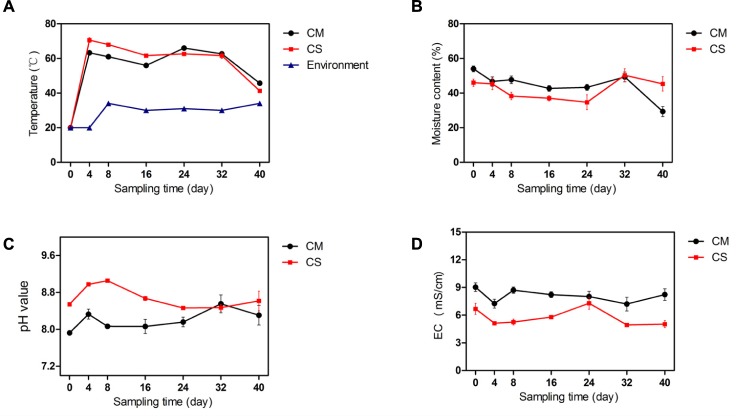
Physicochemical properties of the compost samples. **(A–D)** show the temperature, moisture content, pH, and EC of compost samples, respectively. The data and error bars are the mean and standard deviation of three subsamples. CM, middle layer (∼20 cm depth) of chicken manure compost. CS, middle layer (∼20 cm depth) of chicken manure compost with added maize straw. Environment, environmental temperature.

Compost is a self-heating habitat, in which temperature changes with time. Based on measurement of the compost temperature, both piles of compost entered the thermophilic phase from day 4, with the temperature of the chicken manure compost (CM) reaching 63°C ± 2.5, while the temperature of the chicken manure compost supplemented with maize straw (CS) reached 71°C ± 2.5 (**Figure 1A**), indicating a relatively higher activity of the microbiota in the CS. The thermophilic phases of CM and CS lasted until day 32, when the temperature dropped to below 50°C.

As shown in **Figure 1B**, the initial moisture contents of CM and CS were 54 ± 3.0% and 46 ± 3.6%, respectively. With time the moisture contents of these two composts declined, but the moisture content of CS dropped by a mere 2% compared with a 46% drop in the moisture content of CM (**Figure 1B**), suggesting a water conservation effect of maize straw. The increase in moisture content of the two composts on day 32 occurred as a result of rain.

The composts were alkaline as shown in **Figure 1C**. The pH values in CM and CS tended to rise at the beginning, then decline, and the average pH values in CS were 5% higher than in CM. Compared with lignocellulose-based composts that have an initial EC value below 3.0 (Zhang et al., 2015a), the high initial EC values of 9.0 ± 0.8 in CM and 6.7 ± 1.0 in CS indicate distinct physicochemical properties in composts derived from different feedstocks (**Figure 1D**). When the temperature increased, more nitrogen was presumably released in the form of ammonium, leading to a higher pH and lower EC value in CS compared with CM (**Figure 1**). The physicochemical parameters of the chicken manure composts were similar to the results in a previous pre-experiment (Supplementary Figure S1), indicating the repeatability and consistency of the study results.

### Dominant Bacterial Communities in the Chicken Manure-Based Composts

To understand the composition of the microbial communities in the chicken manure composts, pyrosequencing was performed. After filtering, a total of 1,594,638 quality 16S rRNA sequences were identified (Supplementary Table S2). The diversity index and rarefaction curves suggested that the sequencing depth was sufficient for further analysis (Supplementary Figure S2 and Supplementary Table S2). However, the diversity of the CS samples was significantly higher than that of the CM samples (Pearson analysis *p-*value < 0.0001, Supplementary Figure S2), indicating that the addition of maize straw substantially increased the diversity of the microbiota in the chicken manure compost.

**FIGURE 2:**
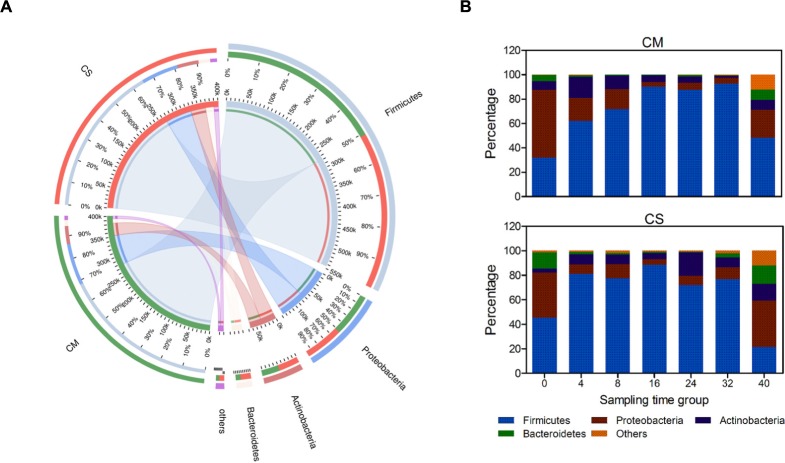
Phylum-level composition of the microbial communities in the compost samples. **(A)** Co-linear relationship map between samples and species at the phylum level; left semicircles represent composition and abundance of the compost samples, while right semicircles represent distribution proportion of the species in different samples. **(B)** Color-coded bar plot represents the average abundances of bacterial phyla across different composting times. Species with relative abundance <1% were classified to the category, Others. CM, middle layer (∼20 cm depth) of chicken manure compost. CS, middle layer (∼20 cm depth) of chicken manure compost with added maize straw.

After taxonomic analysis of the 16S rRNA sequences, a total of 16 phyla were identified, among which four phyla were identified as dominant in that they accounted for nearly 98% of all sequences (**Figure 2A**). *Firmicutes* was the most dominant bacterial phylum, with an abundance of 68%, followed by *Proteobacteria* and *Actinobacteria* (17.4 and 8.7%, respectively), and *Bacteroidetes* was also relatively abundant (3.9%).

**FIGURE 3:**
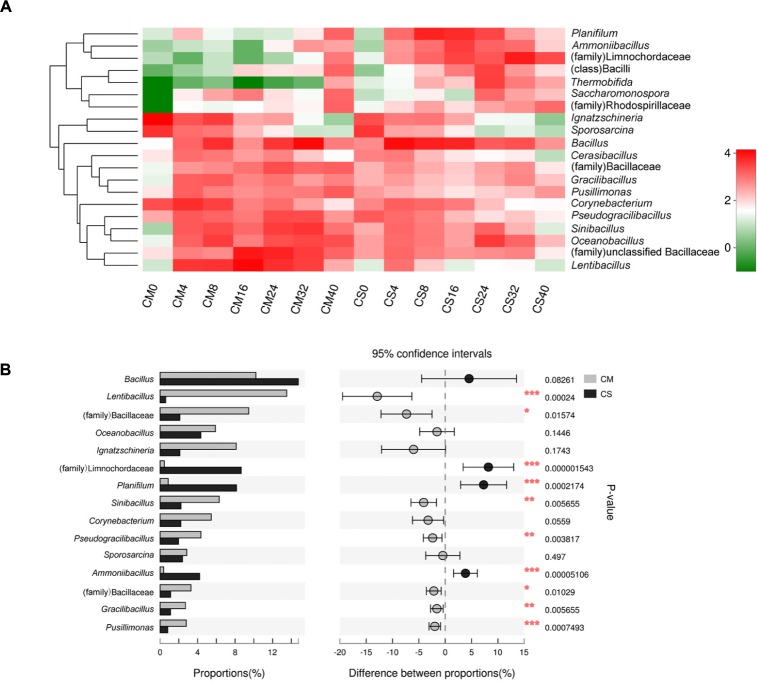
Genus level composition. **(A)** Heatmap of the dominant genera in the compost samples. The top 20 genera were considered, and the color intensity is proportional to the abundance of each genus. Taxa that could not be classified at the genus level but with an abundance greater than 1% are also displayed at the highest taxonomic level. **(B)** The different bacterial genus distributions between the two composts. The top 15 genera were considered, and the difference was considered to be significant between the two data sets at *p* < 0.05 using a *t*-test analysis, which is indicated by asterisks. CM, middle layer (∼20 cm depth) of chicken manure compost. CS, middle layer (∼20 cm depth) of chicken manure compost with added maize straw.

Species succession over time was investigated as shown in **Figures 2**, **3**. Among the four dominant phyla, the abundances of *Firmicutes* and *Proteobacteria* varied considerably (*p* < 0.05 using a *t*-test analysis) along with composting time in both composts. *Actinobacteria* and *Bacteroidetes* varied considerably (*p* < 0.05 using a *t*-test analysis) only in CS and CM, respectively (**Figure 2** and Supplementary Figure S3). In CM, *Proteobacteria* dominated the bacterial community on day 0, with a relative abundance of 56 ± 10% (**Figure 2B**). However, by day 4, the abundance of *Firmicutes* had increased rapidly to 62 ± 8.6%, and on day 16 had reached more than 90 ± 4.5%, at which point *Proteobacteria* accounted for less than 5 ± 2.3%, suggesting this phylum might be derived from the chicken gut microbiota (Shaufi, 2015). These results revealed *Firmicutes* as the dominant bacterial phylum in chicken manure compost, suggesting these organisms may make the biggest contribution to the composting process.

The different succession pattern observed in CS indicated the influence of the feedstock on the microbial composition. CM and CS had similar proportions of *Firmicutes* on day 16 (∼90%), but a different composition pattern before this point (*p* < 0.05 using a *t*-test analysis). In CS, *Firmicutes* dominated the community structure before day 16, with an average abundance of 68%, which was higher than the abundance of 55% in CM (**Figure 2B**). Furthermore, the relative abundance of *Proteobacteria* plummeted to lower than 8% at day 4 in CS, at which point it was 19 ± 0.3% in CM, and the overall abundance of *Proteobacteria* in CS was 2% lower than in CM. Therefore, adding maize straw with a high C:N accelerated the speed of species succession in chicken manure compost during the early stage.

**FIGURE 4:**
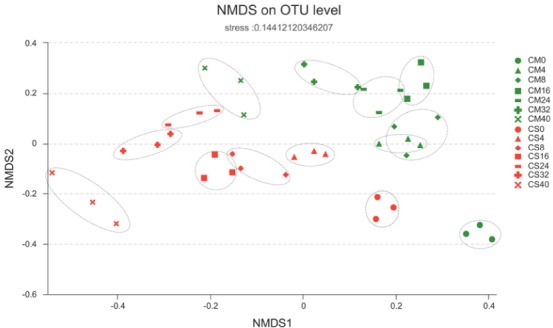
Similarities of the bacterial communities between and within sampling time groups. The distance between samples was calculated by the Bray–Curtis similarity index and plotted with non-metric multidimensional scaling (NMDS), with the OTU similarity set to 0.97. CM, middle layer (∼20 cm depth) of chicken manure compost. CS, middle layer (∼20 cm depth) of chicken manure compost with added maize straw.

Different species succession patterns of the bacterial communities in the two composts were also observed at the genus level (**Figure 3**), and significant impacts of adding maize straw as well as composting time on the distribution of microbiota were observed (**Figures 3B**, **4**). The vast majority of sequences belonging to phylum *Firmicutes* were assigned to the genera *Bacillus* and *Lentibacillus*, accounting for 19.4% of all sequences. The genus *Bacillus* dominated both compost communities, with relative abundances of 10.2 and 14.2% in CM and CS, respectively, but composting time significantly influenced its distribution (*p* < 0.05 using a *t*-test analysis) (Supplementary Figure S4). In CM, this genus peaked at 35.3% at day 32, but at day 16 the abundance was just 1.7 ± 1.1%. In contrast, in CS *Bacillus* dominated the community from day 4, with an abundance of 35 ± 4.2%. Differing from the prominence of *Bacillus* in both compost communities, the compost materials had significant effects on the genus *Lentibacillus* (*p* < 0.05 using a *t*-test analysis) (**Figure 3B**), the abundance of which was 16% in CM and 1.2% in CS. In addition, two other genera belonging to *Firmicutes*, *Planifilum* and *Oceanobacillus*, as well as the family *Limnochordaceae*, were also significantly influenced by the composting materials (*p* < 0.05 using *t*-test analysis). All of them dominated in CS, with the two genera reaching abundances of 20.6 ± 9.5% and 14.1 ± 4.2% at day 8 and day 24, respectively, while the family *Limnochordaceae* had an abundance of 25.8 ± 19.8% at day 32. In addition, the genus *Thermobifida* belonging to the phylum *Actinobacteria* was also significantly influenced by the composting materials (*p* < 10^-5^ using a *t*-test analysis), with a relative abundance of 12.5 ± 1.6% at day 24. Meanwhile, the genus *Ignatzschineria* belonging to *Proteobacteria* was dominant at day 0 with relative abundances of 36.9 ± 12.5% and 8.1 ± 0.8% in CM and CS, respectively, but this decreased sharply to 1% by day 24, and it could hardly be detected after day 32 (**Figure 3**). Members of this genus are autotrophic nitrifiers, could use the nitrogen resource in livestock manure, and are inhibited at the higher temperatures prevalent in composts (Yang et al., 2017).

### Zymogram Assay of Extracellular Proteases

Given that the nitrogen content of chicken manure can reach 2.1% (Ogunwande et al., 2008), microbes proficient in protein degradation may be among the dominant members of its microbial communities. The compositions of the proteases in all samples were determined using native zymogram assays (Supplementary Figure S5A). The results revealed that, during the initial stages of the composting process, proteases were diverse. However, from day 4 in CM and CS, the pattern of bands became clear and apparent in zymogram assays, indicating a change of function in the microbial communities. The compositions of proteases in CM and CS differed throughout the entire composting process, indicating distinct microbial functions in these two composts. In CM, the number and intensity of the protease bands decreased with time, suggesting that high temperature might influence the activities of proteases; the increase at day 40 might be the result of a decline in composting temperature. In contrast, the composition of proteases in CS was similar and varied up to day 24 (Supplementary Figure S5A), suggesting that the dominant functional communities could endure the high temperature and developed with time in this compost.

### Metaproteomics Secreted by the Dominant Microbial Community

**FIGURE 5:**
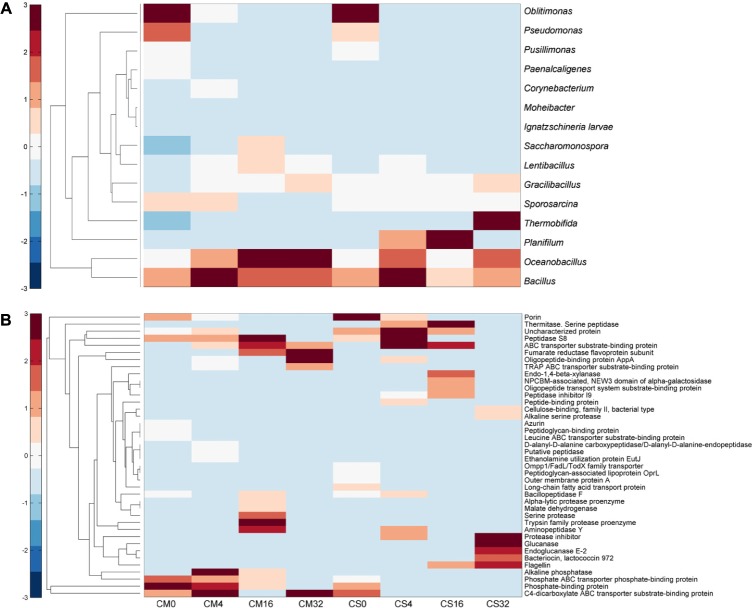
Heatmap of identified proteins of the samples along with composting time. **(A)** Relative abundances of identified proteins belonging to dominant genera. **(B)** Relative abundances of identified extracellular proteins. CM, middle layer (∼20 cm depth) of chicken manure compost. CS, middle layer (∼20 cm depth) of chicken manure compost with added maize straw. Samples from days 0, 4, 16, and 32 were chosen for performing metaproteomics analysis. The software Matlab (version R2014a) was used to conduct this analysis.

To further understand the functions of the dominant microbial communities in chicken manure composts, the LTQ-Orbitrap technique was used to identify the origin of major secreted proteins (Makarov et al., 2006). As shown in **Figure 5**, samples collected at days 0, 4, 16, and 32 from CM and CS were investigated, and the genera with relative abundances in the top 20 at each sampling time were used as reference databases. According to the metaproteomics data, a total of 621 proteins belonging to bacteria were identified, of which 68 were extracellular proteins (**Figure 5** and Supplementary Table S3).

More than half of the identified proteins were secreted by *Bacillus*, *Oceanobacillus*, and *Planifilum*, which also dominated at the genomic level. Besides, proteins derived from other dominant genera were identified, and the pattern of metaproteomics matched well with the 16S rRNA sequencing data. For instance, as shown in **Figure 5**, proteins belonging to several microbes prevalent in livestock manure, such as *Pseudomonas* and *Oblitimonas*, were identified at day 0 at relatively high abundances; at the same time they were also predominant in 16S rRNA sequencing data (Akinde and Obire, 2008; Gupta et al., 2014). However, after 4 days of fermentation, their proteins were hardly detected, but were replaced by proteins secreted by *Bacillus* and *Oceanobacillus*. It is worth noting that at day 16, distinct metaproteome patterns appeared in CM and CS; that is, in addition to the dominant members at day 4, proteins belonging to the genera *Lentibacillus* and *Saccharomonospora* also dominated the CM metaproteome, while proteins secreted by *Planifilum* exclusively dominated the CS metaproteome with a relative abundance of 68%. This difference continued to day 32, at which time *Thermobifida* was most predominant in CS with a relative abundance of 36 ± 2.3%.

In the bacterial metaexoproteomics of the compost samples, most of the identified proteins were classified to the function category transportation, accounting for 32% and 19% in CM and CS metaexoproteomics, respectively. These transportation-related proteins were widely distributed among the composting stages, and 38 ± 6.3% and 60 ± 9.3% were secreted in CM and CS, respectively, by the species *Oblitimonas alkaliphila* at day 0. However, they only appeared at day 0 and day 4; none of them were detected after this. Moreover, the extracellular proteins secreted overall by this species, including proteins with other functions, were only detected at day 0 and day 4 as well (Supplementary Figure S6 and Supplementary Table S3). The sharp decrease in the abundance of this genus in the high-throughput sequencing data also supported this conclusion (Supplementary Figure S7). These results suggest that *Oblitimonas* might be derived from the chicken manure, and its growth was inhibited by the increasing temperature. In addition, 8 and 10% of the identified transportation-related proteins belonged to the genera *Bacillus* and *Oceanobacillus*, respectively.

The compost microbiota also displayed significant nitrogen resource utilization; that is, 21 and 34% of the identified proteins were classified to the function amino acid metabolism in CM and CS, respectively. Proteins belonging to *Bacillus* and *Saccharomonospora* dominated the CM group, reaching a peak at day 16 with relative abundances of 8 ± 3.2% and 39 ± 5.6%, respectively. Meanwhile in CS, *Planifilum* and *Thermobifida* dominated the group, whose proteins accounted for 41 ± 4.8% and 31 ± 6.9% of all identified proteins at day 16 and day 32, respectively. The different distribution patterns might indicate that these microbes occupy different niches. These four genera were identified previously in composts, and are known to secrete thermostable proteases (Hatayama et al., 2005; Chen and Wilson, 2007; Jani and Patel, 2014; Jugran et al., 2016), indicating their importance in the degradation of compost feedstock.

Microbiota in CS displayed a stronger carbohydrate metabolism than those in CM, as five glucoside hydrolases belonging to *Planifilum* and *Thermobifida* were identified, including one xylanase (A0A1I2PGX8) and one endoglucanase (P26222). Besides, carbohydrases secreted by *Planifilum* accounted for 23 ± 2.0% at day 16 in CS, and at day 32 41 ± 2.2% of the proteins were classified as glucoside hydrolases of *Thermobifida*. Just one protein involved in carbohydrate metabolism was identified, which belonged to the genus *Sporosarcina*, and it only appeared at day 16 with a relative abundance of 6 ± 0.9%. Maize straw contains lignocellulose, and five different types of glycoside hydrolase were identified in CS samples, suggesting the addition of maize straw altered the function of the dominant microbial communities, resulting in a shift from protein degradation to lignocellulose degradation, which could be supported by the higher endoglucanase activities in CS samples compared to CM samples (Supplementary Figure S5B).

In addition to the above dominant metabolism functions, one type of antibacterial protein belonging to *Thermobifida* was detected, accounting for 13 ± 1.2% at day 32 in CS. suggesting that microbes from this genus defended themselves and competed with other microbes by secreting antibacterial proteins.

## Discussion

### Species Succession Was Driven by High Temperature in the Two Chicken Manure Composts

This study revealed the composition of the dominant functional microbiota in chicken manure composts. In the two chicken manure composts, *Firmicutes*, *Proteobacteria*, *Actinobacteria*, and *Bacteroidetes* were the most predominant phyla in the compost microbiota. In addition, among the four dominant phyla, three displayed a significant correlation with temperature (Spearman correlation analysis *p* < 0.05, Supplementary Figure S8), while other factors such as moisture, pH, and EC had no obvious impact on them. As mentioned before in previous studies, temperature can influence the structure and diversity of compost microbial communities, thereby affecting the species succession process in composts (Miyatake and Iwabuchi, 2005; Zhang et al., 2015a).

Temperature was the immediate parameter characterizing the different stages of aerobic composting. The investigation of these two chicken manure composts revealed that both piles had a good start, entering the thermophilic stage at day 4 (**Figure 1**). The difference between them was that in the CS, the temperature in the initial stage was 8°C higher than that in CM, and the average temperature was higher by 2°C compared with CM. This suggested that, to a certain degree, adding biomass with a high C:N ratio such as maize straw can enhance the activity of the microbial community in chicken manure compost.

According to the previous studies, when temperature reached 50°C under aerobic condition, most bacterial pathogens and protozoa were inactivated within 24 h (Bicudo and Goyal, 2003). Hence in this study, since the inner temperature of the compost maintained over 50°C more than 28 days, the harmless treatment of the composting materials was considered to be achieved. In addition, the abundances of *Bacteroidetes* and *Proteobacteria* sharply decreased with time (**Figure 2**). Since the two phyla contain diverse plant and human pathogens (Tsolis, 2002; Romero et al., 2010), maintaining a high temperature in composts could therefore inhibit the growth and spread of pathogens, and contribute to the harmless treatment of feedstock. Composts with temperatures under 50°C cannot achieve efficient species succession (Cui et al., 2015; Zhang et al., 2016). Therefore, to realize the goal of harmless treatment of feedstock, high temperatures in the composts were needed.

### *Firmicutes* Dominated the Chicken Manure Compost Microbiota

In the chicken manure-based composts, *Firmicutes* was the most dominant bacterial phylum with an abundance of 68% (**Figure 2A**). *Firmicutes* can secrete various proteases and pectinases, and can degrade indigestible carbohydrates such as cellulose (Stanley et al., 2013). It can thus can play a leading role in chicken manure composts with a high nitrogen content (Shimogaki et al., 2014; Singh et al., 2016). Besides, this phylum also dominated the cecal microbiomes of feral chickens (Ferrario et al., 2017). This could explain why the relative abundance of this phylum was below 25% in regular maize straw compost (Zhang et al., 2015a), but much higher in the chicken manure compost added with maize straw used in the present study. This is consistent with a fermenter maize straw compost based on cow dung in which *Firmicutes* was the dominant bacterial phylum (Zhang et al., 2015b). In addition, our previous work, similar to this research, also supported this conclusion. In that study, *Firmicutes* dominated the compost communities as well (Supplementary Figure S9).

The genus *Bacillus*, belonging to *Firmicutes*, was the dominant bacterium in both composts with a relative abundance of 16% (**Figure 3**). *Bacillus* is well known for the production of thermostable proteases (Jellouli et al., 2011; Anandharaj et al., 2016), and strains belonging to this genus have been isolated from manure compost previously (Charbonneau et al., 2012). *Bacillus* might therefore play an important role in the degradation of nitrogen sources such as chicken manure. The high abundance of these organisms suggests they may be the source of the highly active secreted proteases detected in the zymogram assays (Supplementary Figure S5A). The identification of diverse proteases secreted by this genus also proved its substantial contribution to feedstock degradation (**Figure 5**).

### The Composition of the Dominant Microbial Community Was Altered by Adding Maize Straw to the Chicken Manure Compost

In chicken manure composts, the structure of the dominant microbiota (**Figure 3B**) was significantly influenced by adding maize straw to the composts, despite the fact that no significant influence of composting materials was found at the phylum level. Among the 20 dominant genera, 13 were significantly affected by adding maize straw. For instance, the genus *Lentibacillus* was most dominant in CM with a relative abundance of 16%, but only accounted for nearly 1% in CS. Members of this genus are halophilic bacteria that can grow over a wide pH and temperature range (Sun et al., 2016). Unlike this genus, adding maize straw positively impacted *Limnochordaceae*, *Planifilum*, *Oceanobacillus*, and *Thermobifida*, significantly increasing their abundances in CS. All four taxa are thermophilic bacteria that have been found in different composts before (Cui et al., 2015; Sakai et al., 2015; Watanabe et al., 2015). However, further research is needed to reveal the mechanisms underlying reductions or increases in the abundances of these microorganisms upon the addition of maize straw.

The exception *Bacillus*, was the most dominant genus in terms of relative abundance in both piles. This genus is very common in manure-based composts (Maeda et al., 2011; Mc et al., 2011), and is also prevalent in lignocellulose-based composts (Zhang et al., 2015b), indicating its formidable viability in different habitats.

Most notably, adding maize straw to chicken manure compost significantly increased the function of carbohydrate metabolism (*p*-value < 0.001, Supplementary Figure S10). As one type of major industrial crop biomass, maize straw contains high concentrations of lignocellulose, which can be degraded by microorganisms with the capacity to degrade carbohydrates (Wilson, 2011). While another industrial crop biomass, wheat straw was an inefficient composting material for rapid composting, mainly due to that the wheat straw is surrounded with a layer of wax, which impeds the degradation of microbes toward the wheat straw (Zhang et al., 2016). In CS, the relative abundance of the phylum *Actinobacteria* increased slightly, and three glycoside hydrolases secreted by *Thermobifida fusca* were detected (**Figures 2**, **5**). The genus *Thermobifida* was reported to be one of the most dominant genera in maize straw-based composts, in which it plays an important role in cellulose degradation (Zhang et al., 2015a,b). Here the identification of its enzymes together with the increase in *Actinobacteria* indicates that the composting material can alter the functions of microbiota in composts.

### Identification of a Dominant Microbiota That Participates in Nitrogen Source Utilization in Chicken Manure Composts

A characteristic of chicken manure is its high content of nitrogen source. Therefore, microbes appearing in chicken manure composts should have the ability to degrade and utilize this nitrogen resource. In this study different kinds of proteases were identified in the metaproteome of the compost communities (**Figure 5**), proving their capacity to degrade chicken manure. Function prediction of the compost microbiota based on 16S rRNA sequences also supported this result: that amino acid transport and metabolism were the most dominant functions in both composts (Supplementary Figures S11, S12). In chicken manure composts diverse microbes with the ability to utilize nitrogen sources had been identified in previous research, including members belonging to *Firmicutes* (Maeda et al., 2011). In this work, in chicken manure composts the dominant microbiota was identified, mainly including *Bacillus*, *Lentibacillus*, *Limnochordaceae*, *Planifilum*, and so on. Prominent features of this dominant microbiota are the ability to utilize nitrogen sources and facilitation of amino acid transport and metabolism, characteristics that are distinctly different from those of microbiota in lignocellulose-based composts, where microbes capable of degrading lignocellulose are more dominant (Zhang et al., 2014).

These findings highlight the distinct impact of different composting materials on the composition of the dominant functional microbiota in chicken manure composts. *Firmicutes* was the most dominant bacterial phylum, members of which such as *Bacillus* play an important role in feedstock degradation by secreting various proteases. However, the addition of maize straw to chicken manure compost altered the functional microbiota structure, and *Bacillus*, *Limnochordaceae*, *Planifilum*, and *Thermobifida* became the dominant functional bacteria. In addition, the ability to metabolize carbohydrates was significantly enhanced with the addition of maize straw. The abundance of the phylum *Proteobacteria* decreased sharply after the start of composting, which may be the result of high temperatures that inhibit its growth. Adding maize straw to chicken manure composts may therefore accelerate species succession to establish a stable dominant functional microbiota, and more efficiently render harmless feedstocks that can subsequently be used as agricultural fertilizers.

## Data Availability Statement

The sequencing data datasets generated for this study can be found in the NCBI Sequence Read Archive under accession codes SRP117004 (https://trace.ncbi.nlm.nih.gov/Traces/sra/sra.cgi?study=SRP117004) and SRP117012 (https://trace.ncbi.nlm.nih.gov/Traces/sra/sra.cgi?study=SRP117012).

## Author Contributions

LZ conceived the study design, conducted the sampling, performed sequencing and data analysis and wrote the paper. LL conducted the sampling and analyzed the data. XP and ZS conducted the sampling. XF, BG, and JL edited and reviewed the manuscript. LW conceived the study design and edited the paper. All authors approved the final submitted manuscript.

## Conflict of Interest Statement

The authors declare that the research was conducted in the absence of any commercial or financial relationships that could be construed as a potential conflict of interest.

## Footnotes

1 http://drive5.com/uparse/

2 http://www.arb-silva.de
