# Supplementary material for: Enhanced Growth and Activities of the Dominant Functional Microbiota of Chicken Manure Composts in the Presence of Maize Straw
Source: Front Microbiol. 2018 May 29;9:1131. doi: 10.3389/fmicb.2018.01131 (PMC5986910; doi:10.3389/fmicb.2018.01131)
Supplement: Supplementary file 1 [file Data_Sheet_1.pdf]

Supplementary Material

Enhanced growth and activities of the dominant functional microbiota of chicken manure composts in the presence of maize straw

Lili Zhang[1, 3], Lijuan Li[1], Xiaoguang Pan[1], Zelu Shi[1], Xihong Feng[2], Bin Gong[3], Jian Li[4] and Lushan Wang[1*]

[1]State Key Laboratory of Microbial Technology, Shandong University, Jinan, China;
[2]Lu Qing Seed Co. Ltd., Jinan, China;
[3]School of Computer Science and Technology, Shandong University, Jinan, China;
[4]Laboratory of Antimicrobial Systems Pharmacology, Monash Biomedicine Discovery Institute, Department of Microbiology, Monash University, Australia

[*]**Correspondence:** Prof. Lushan Wang, lswang@sdu.edu.cn

Table S1. Sample origin of the compost samples.[a]

Sample origin	Sample name			Sampling time
Chicken manure compost	CM0-1	CM0-2	CM0-3	day 0
(CM)	CM4-1	CM4-2	CM4-3	day 4
	CM8-1	CM8-2	CM8-3	day 8
	CM16-1	CM16-2	CM16-3	day 16
	CM24-1	CM24-2	CM24-3	day 24
	CM32-1	CM32-2	CM32-3	day 32
	CM40-1	CM40-2	CM40-3	day 40
Chicken maure ompost	CS0-1	CS0-2	CS0-3	day 0
added maize straw (CS)	CS4-1	CS4-2	CS4-3	day 4
	CS8-1	CS8-2	CS8-3	day 8
	CS16-1	CS16-2	CS16-3	day 16
	CS24-1	CS24-2	CS24-3	day 24
	CS32-1	CS32-2	CS32-3	day 32
	CS40-1	CS40-2	CS40-3	day 40

[a]Abbreviations: CM, chicken manure compost; CS, chicken manure compost added maize straw; number means the day on which the samples were collected.

Table S2. Overview of pyrosequencing data.

	sequence information					diversity index					
Sample	Sequence number	Base number	Mean length	Min length	Max length	ace	chao	coverage	shannon	simpson	sobs
CM0_1	41574	18588461	447.1175	328	500	482.244	485.2174	0.997183	3.448934	0.085224	384
CM0_2	41368	18477884	446.671	336	499	505.4037	519.8	0.997311	3.468079	0.100607	412
CM0_3	43360	19420983	447.9009	218	495	546.5263	515.0909	0.997646	3.16032	0.175228	374
CM4_1	43589	19455439	446.3383	341	499	635.6364	591	0.997	3.604612	0.061043	421
CM4_2	31890	14240915	446.5637	347	505	529.7883	529	0.996229	4.056009	0.041767	426
CM4_3	32474	14564607	448.5006	396	497	448.2467	443.9184	0.99663	3.657622	0.063583	364
CM8_1	38173	17130326	448.755	276	505	407.9521	409.0244	0.997285	3.144001	0.126477	324
CM8_2	37077	16669346	449.5872	381	504	456.7588	434.7759	0.996884	2.735043	0.125736	337
CM8_3	44631	19963691	447.3055	388	503	427.9652	448.8857	0.997617	3.605571	0.059965	354
CM16_1	36905	16604811	449.9339	396	460	475.1167	389.037	0.997228	1.936235	0.278359	234
CM16_2	33838	15197519	449.1258	378	501	423.5593	439.3	0.997069	2.939673	0.14869	265
CM16_3	32878	14779174	449.5156	343	461	350.7949	345.025	0.997303	2.354849	0.234604	268
CM24_1	35397	15882877	448.7069	339	487	429.4635	417.6538	0.997047	3.563716	0.058055	349
CM24_2	40526	18229406	449.82	396	502	449.7277	420.8125	0.997335	2.90384	0.118897	290
CM24_3	43292	19471477	449.7708	304	495	337.9037	334.5526	0.997992	2.778539	0.124802	271
CM32_1	35759	16076821	449.5881	327	498	452.3197	429.5333	0.997352	3.164701	0.086215	299
CM32_2	40373	18162329	449.8633	403	499	357.7529	341.4348	0.997513	2.049942	0.368927	271
CM32_3	40041	18018136	449.9922	237	496	533.6996	452.1538	0.996991	2.800169	0.127863	304
CM40_1	40463	18128936	448.0374	356	502	445.1734	454.0256	0.997283	3.905407	0.047514	373
CM40_2	35878	15850106	441.7779	292	489	481.8637	497.3488	0.996181	4.057731	0.037199	400

CM40_3	32434	14315562	441.3752	343	468	510.9084	510	0.996622	3.953163	0.044777	411
CS0_1	38688	17334939	448.0702	340	496	645.6845	648.3088	0.994888	4.263906	0.034533	525
CS0_2	41776	18663336	446.7478	304	503	809.9758	842.28	0.995134	4.595649	0.024881	679
CS0_3	39039	17447290	446.9195	358	502	754.9659	755.686	0.99463	4.268145	0.044629	624
CS4_1	37674	16841666	447.0368	358	500	723.0439	763.1194	0.994121	3.926731	0.059984	578
CS4_2	39887	17795114	446.1382	392	499	807.4153	790.7238	0.995975	4.320886	0.05552	702
CS4_3	44524	19948112	448.0305	380	503	777.2996	781.2593	0.995359	4.16122	0.052928	632
CS8_1	44184	19763633	447.3029	340	499	784.0233	801.5556	0.995784	3.664823	0.116584	658
CS8_2	40862	18205637	445.5395	279	501	780.9182	769.8542	0.994627	4.02138	0.065571	655
CS8_3	42577	18995272	446.1393	341	475	789.8523	800.6395	0.994313	4.201898	0.038959	649
CS16_1	31710	14142306	445.9888	389	499	720.4147	745.1549	0.993468	3.900827	0.05498	566
CS16_2	33898	15118738	446.0068	389	499	777.1499	709.1186	0.993879	3.090321	0.110478	477
CS16_3	34760	15456011	444.6493	397	505	738.5787	761.0143	0.993481	3.764315	0.071738	563
CS24_1	39643	17582231	443.5141	325	500	531.4783	538.3889	0.995742	3.781343	0.049009	417
CS24_2	31647	14142450	446.8812	366	499	611.1465	546.4118	0.995036	3.504665	0.061437	392
CS24_3	36116	16115390	446.2119	366	502	620.2928	626.75	0.994821	3.330666	0.072381	438
CS32_1	30437	13463672	442.3456	317	497	677.6883	664.241	0.992931	4.567021	0.023017	552
CS32_2	44189	19296419	436.6792	389	491	718.9072	742.0448	0.996197	4.298432	0.039648	613
CS32_3	38391	17091939	445.2069	341	493	573.9452	564	0.994411	4.333695	0.027741	447
CS40_1	31039	13731795	442.4046	268	494	728.2293	725.04	0.99515	5.083729	0.012638	633
CS40_2	35563	15651680	440.1114	287	502	678.9463	707.9672	0.995003	4.450016	0.026749	553
CS40_3	36114	15949963	441.656	321	488	743.7269	771.0159	0.995031	4.935164	0.016025	642

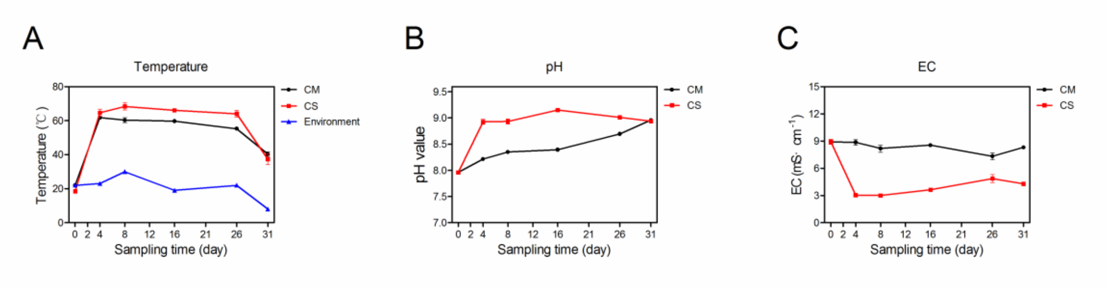

Fig. S1. Physicochemical properties of compost samples. This experiment was done in June, 2015. The data and error bars are the mean and standard deviation of three subsamples. A, B and C show the temperature, pH and EC of compost samples, respectively. CM, middle layer (~20 cm depth) of chicken manure compost. CS, middle layer (~20 cm depth) of chicken manure compost with added maize straw. Environment, environmental temperature.

A

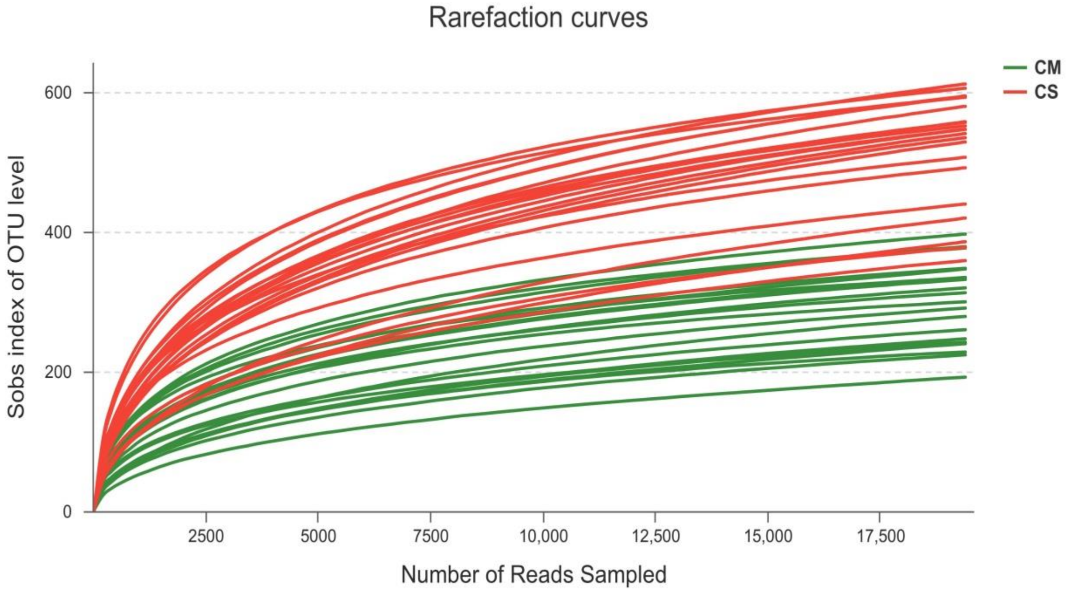

B

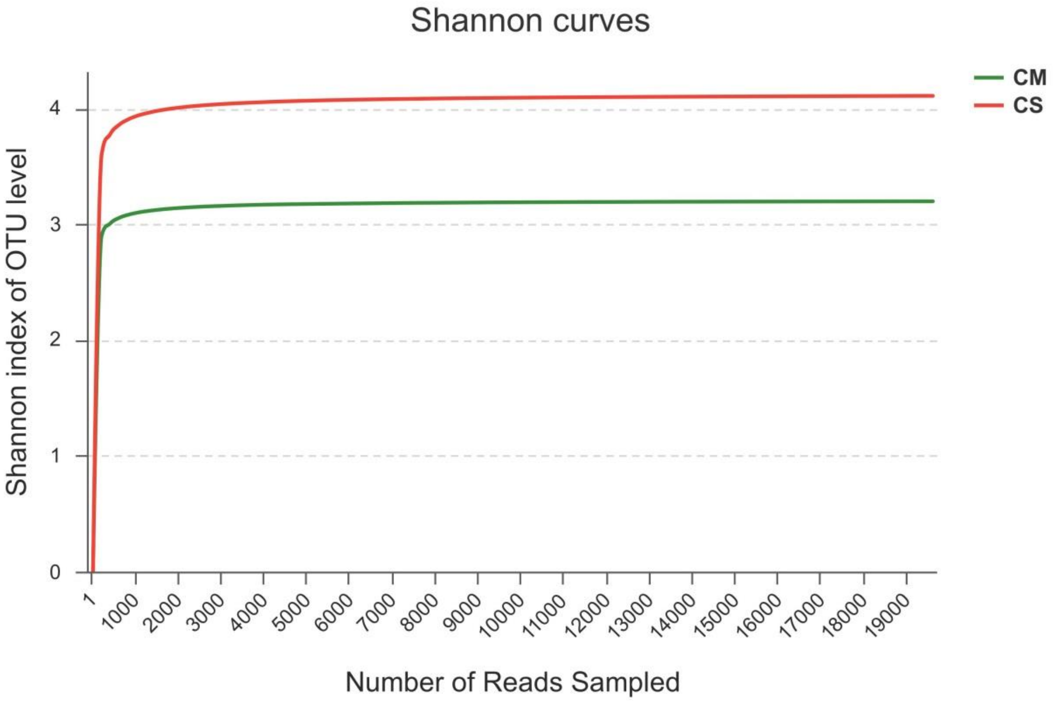

Fig. S2. The average rarefaction curve representing variation of the Sobs (A) and Shannon diversity index (B) at increasing sequencing depth of compost samples.

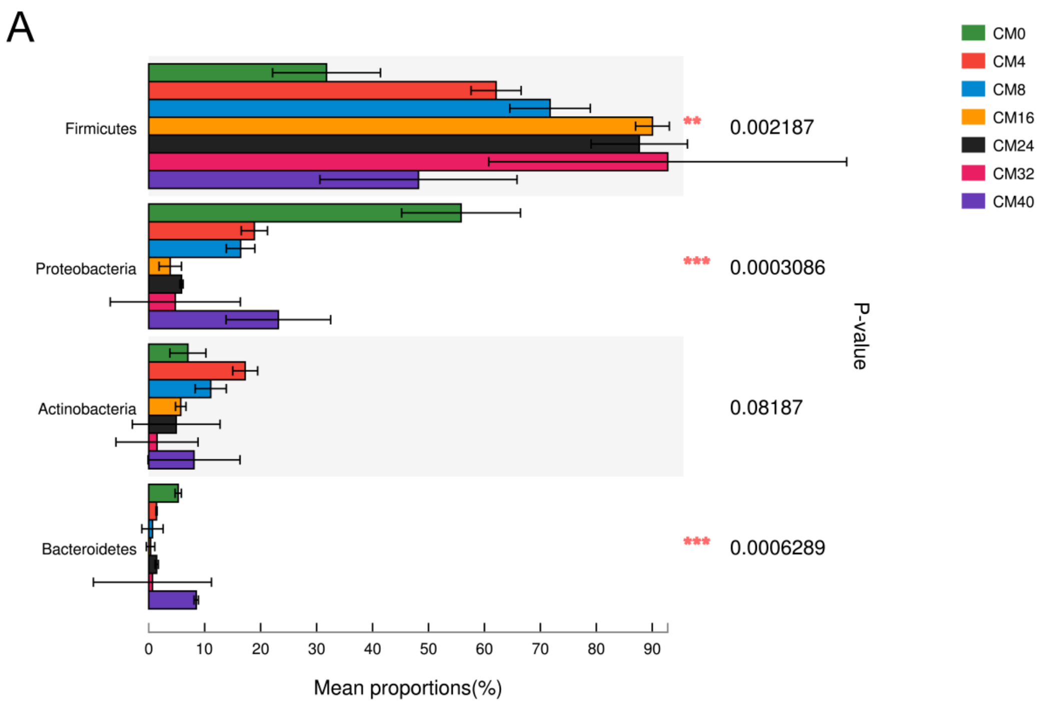

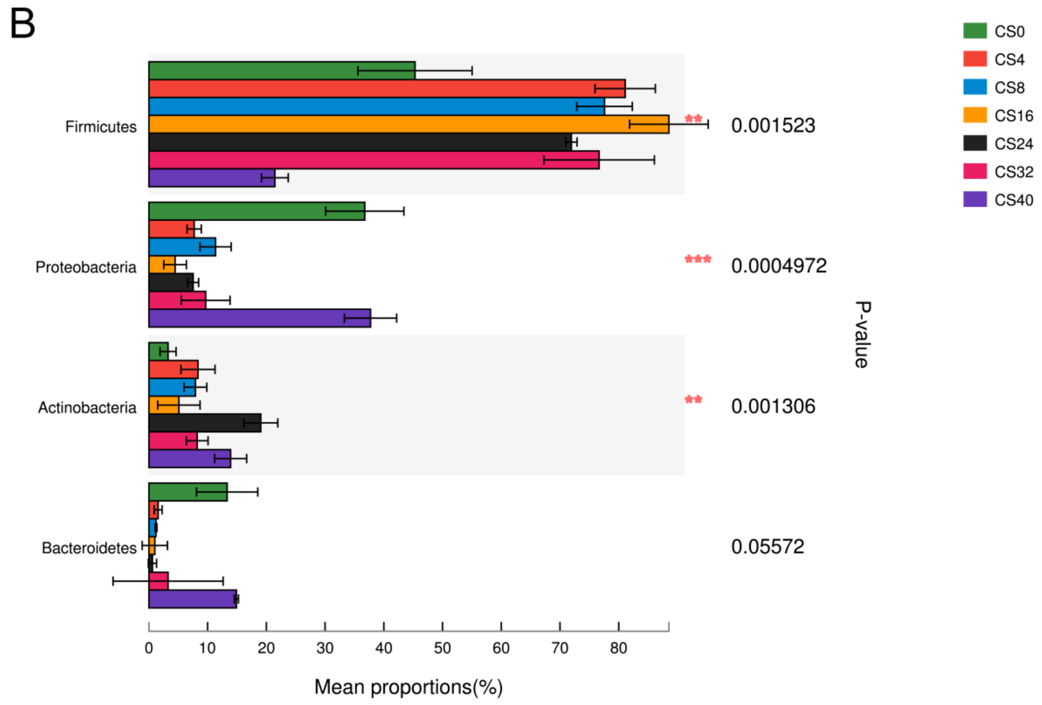

Fig. S3. The phylum level composition of the microbiota in CM (A) and CS (B). One-way ANOVA analysis was applied on the computation of the abudance changes.

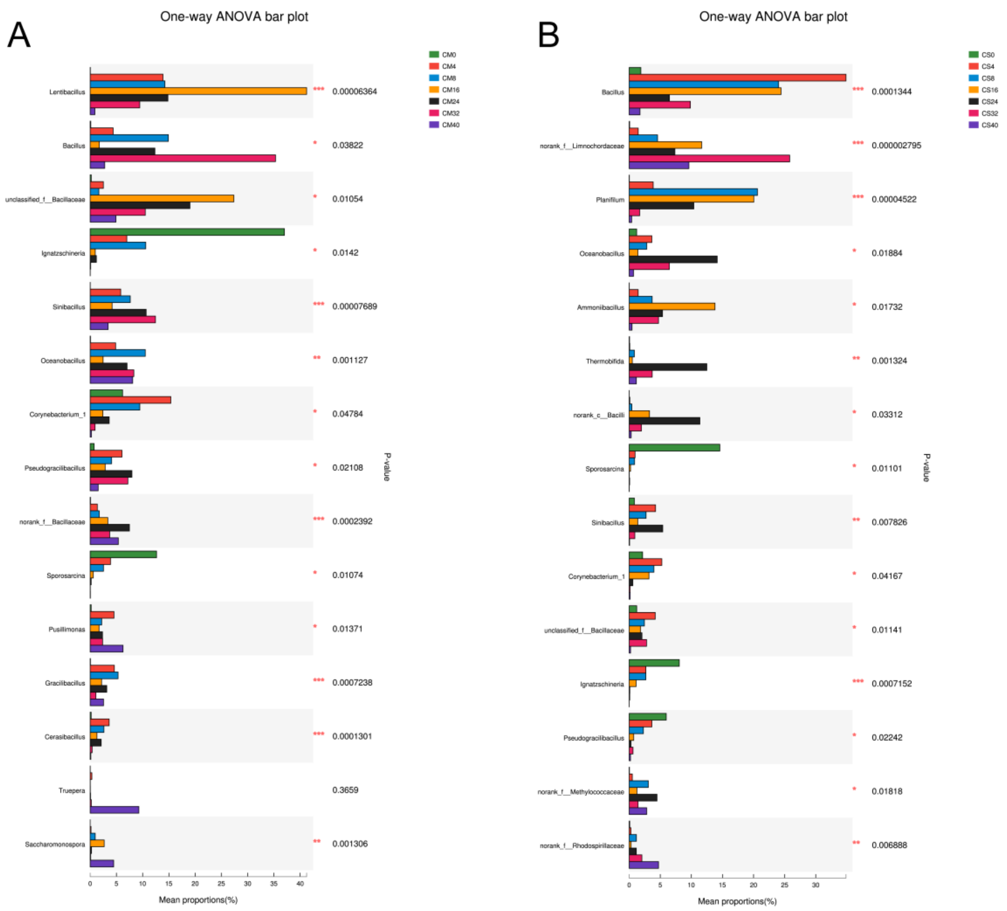

Fig. S4. The genus level composition of the microbiota in CM (A) and CS (B). One-way ANOVA analysis was applied on the computation of the abudance changes.

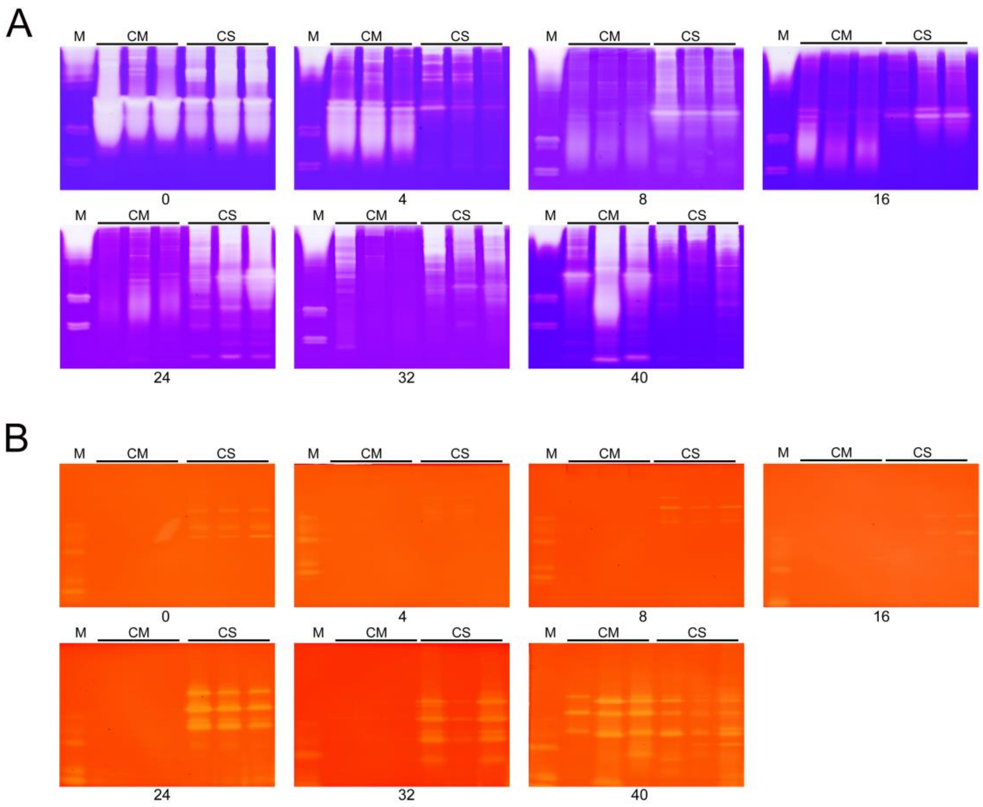

Fig. S5. Zymogram of the proteases (A) and extracellular endoglucanases (B) from samples in CM and CS. CM, middle layer (~20 cm depth) of chicken manure compost. CS, middle layer (~20 cm depth) of chicken manure compost with added maize straw. M, the crude enzymes of *Aspergillus niger AN76*. Numbers indicate the sampling day.

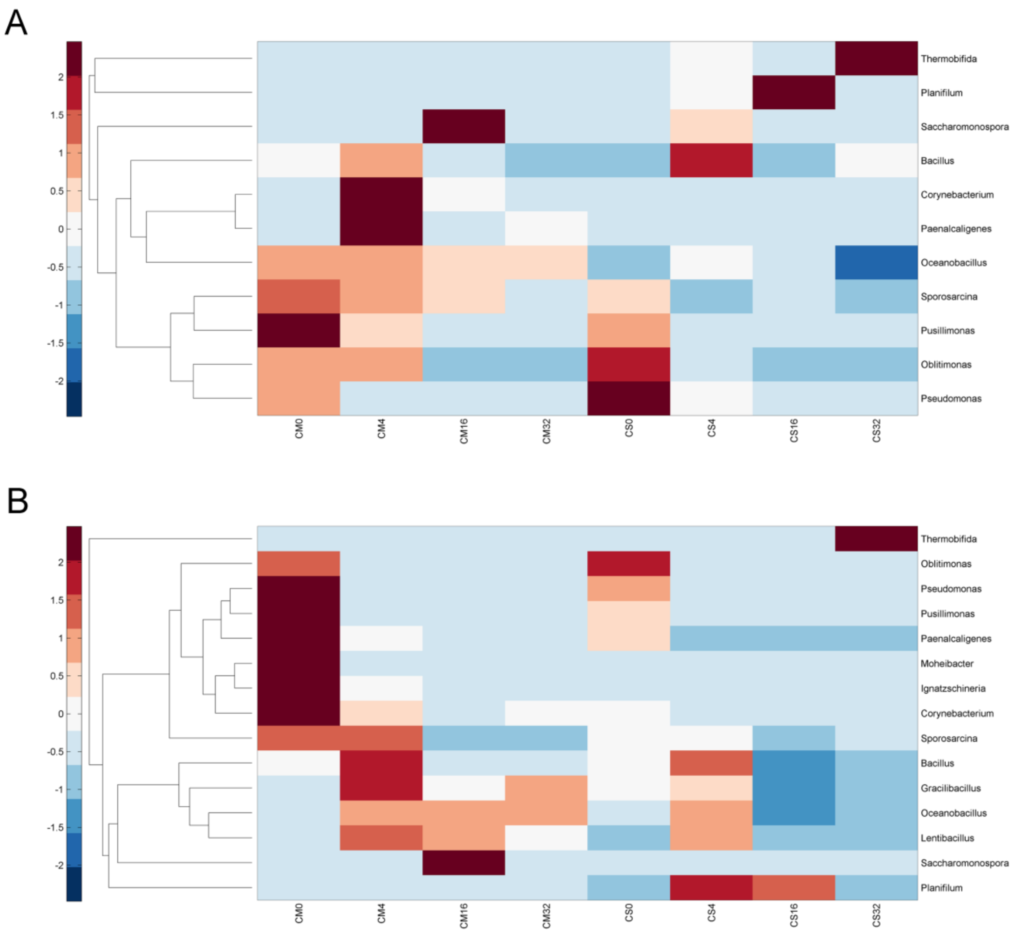

Fig. S6. Heatmap of the relative abundance of predicted extracellular proteins secreted by dominant genera of CM (A) and CS (B) samples. Color represented the relative abundance, which increased from green to red.

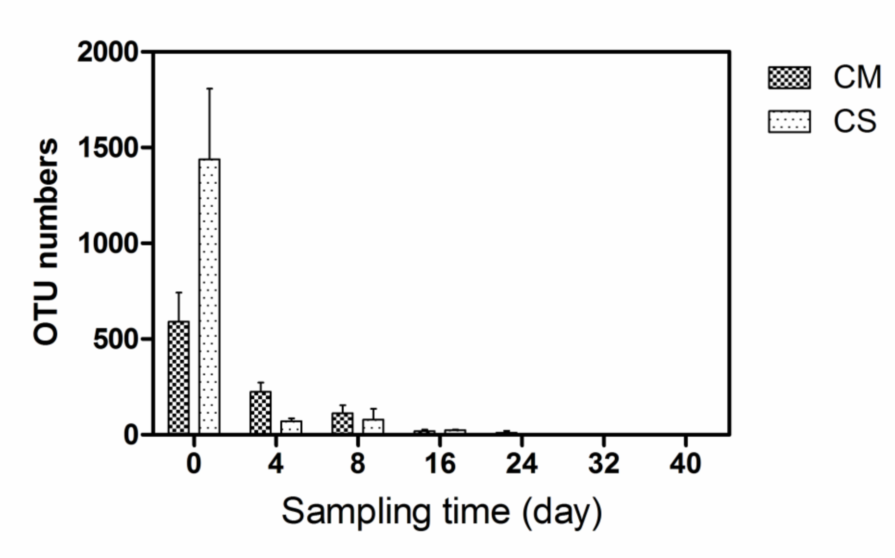

Fig. S7. The detected OTU numbers of the genus *Oblitimonas* in CM and CS samples along with composting time.

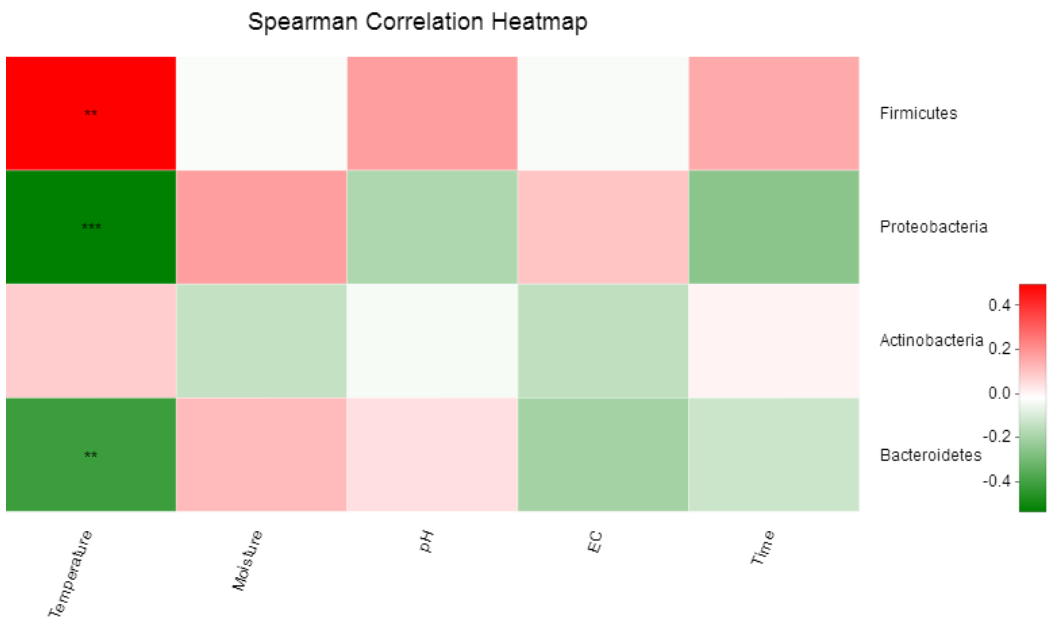

Fig. S8. Heatmap of correlation between microbial communities and different environmental factors. Color represented the correlation degree, the degree increased from green to red. Temperature, moisture, pH, EC and composting time were considered in this analysis as environmental factors. The four dominant phyla in Fig. 2 were chosed.

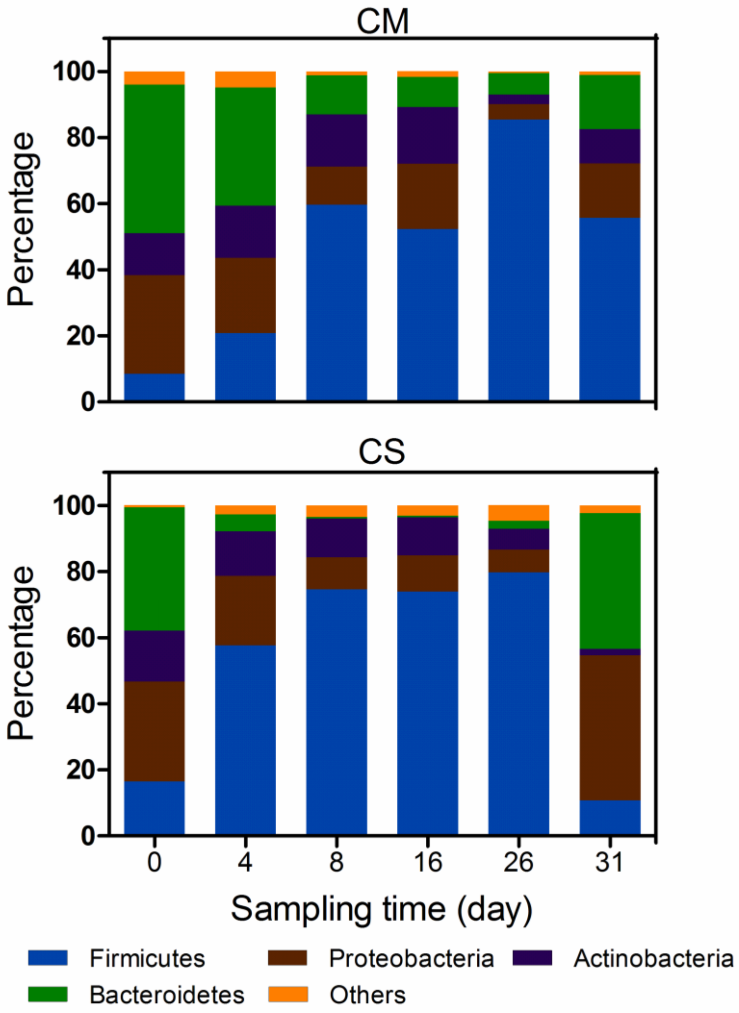

Fig. S9. Phylum level composition of microbial communities of compost samples. Color coded bar plot represents the average abundance of bacterial phyla across different composting time. CM, middle layer (~20 cm depth) of chicken manure compost. CS, middle layer (~20 cm depth) of chicken manure compost with added maize straw. Others, phyla with relative abundances less than 1% were classified into this category. Sequencing data generated in this study have been deposited at the NCBI Sequence Read Archive under accession codes SRP078780 and SRP078783.

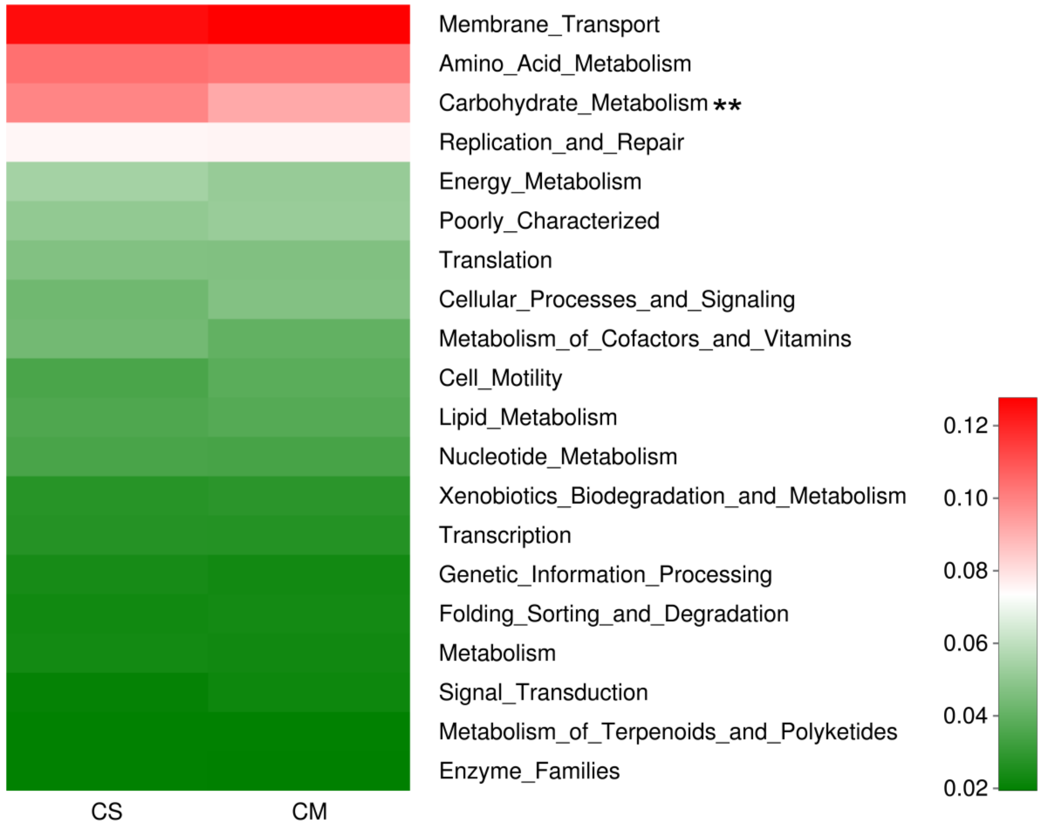

Fig. S10. Heatmap of the relative abundance of predicted functions of CM and CS samples based on the PICRUSt. Color represented the relative abundance, which increased from green to red. Asterisk represents significant difference of relative abundance of corresponding function between these two piles. Functions with relative abundance more than 1% were listed here.

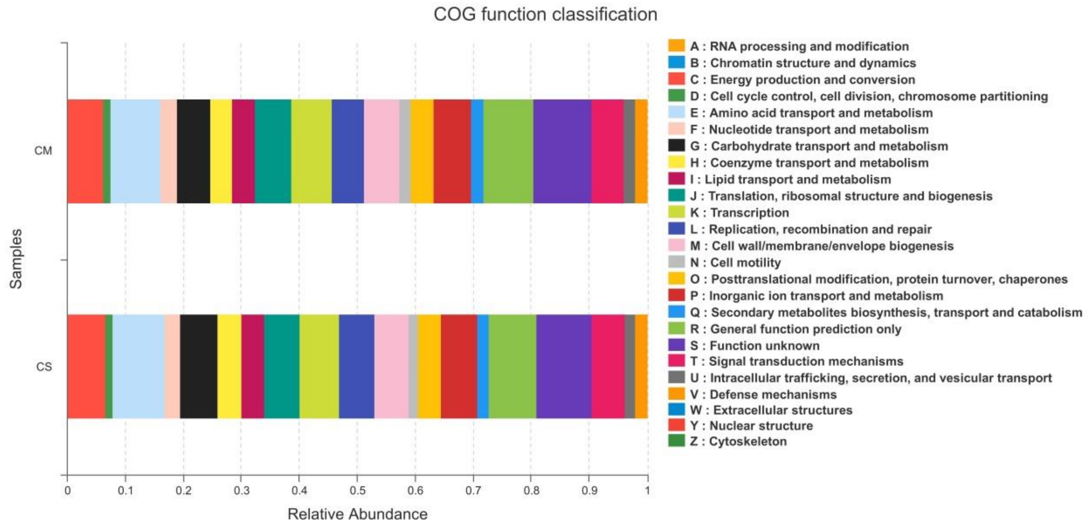

Fig. S11. Predicted functions of CM and CS samples based on the PICRUSt.

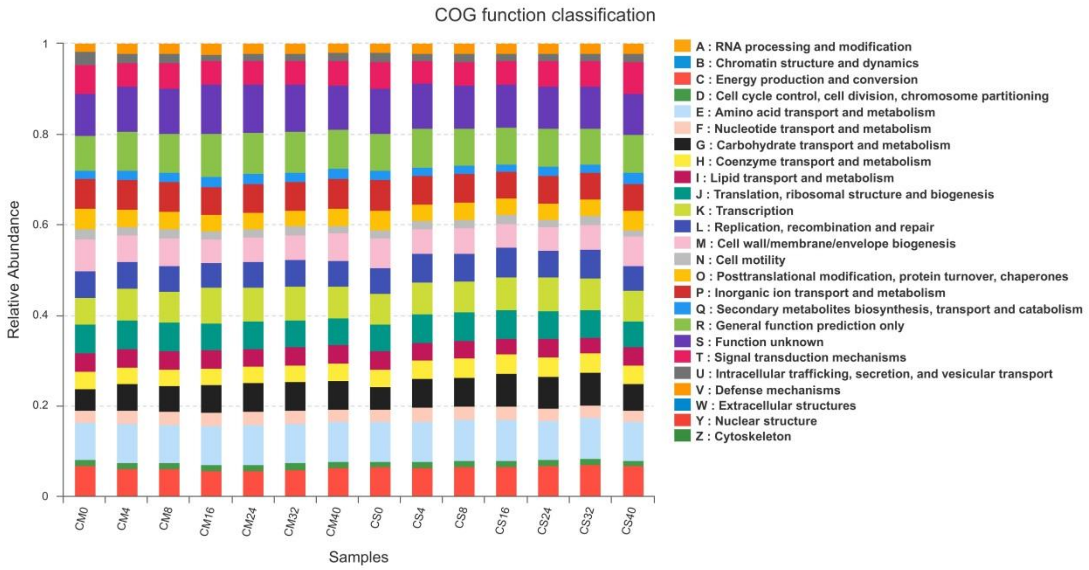

Fig. S12. Predicted functions of CM and CS samples at different sampling times based on the PICRUSt.
